# Risk analyses of nocturia on incident poor sleep and vice versa: the Nagahama study

**DOI:** 10.1038/s41598-023-36707-y

**Published:** 2023-06-11

**Authors:** Hiromitsu Negoro, Kazuya Setoh, Arinobu Fukunaga, Takahisa Kawaguchi, Satoshi Funada, Takayuki Yoshino, Koji Yoshimura, Bryan J. Mathis, Yasuharu Tabara, Fumihiko Matsuda, Osamu Ogawa, Takashi Kobayashi

**Affiliations:** 1 Department of Urology, University of Tsukuba, 1-1-1 Tennodai, Tsukuba, Ibaraki 305-8575 Japan; 2 Department of Urology, Kyoto University Graduate School of Medicine, Kyoto, Japan; 3 Center for Genomic Medicine, Kyoto University Graduate School of Medicine, Kyoto, Japan; 4 Graduate School of Public Health, Shizuoka Graduate University of Public Health, Aoi-Ku, Shizuoka, Japan; 5 Department of Urology, Shizuoka General Hospital, Shizuoka, Japan; 6 International Medical Center, University of Tsukuba Affiliated Hospital, Ibaraki, Japan

**Keywords:** Health care, Urology

## Abstract

Cross-sectional relationships between nocturia and sleep problems have been well evaluated but the risk association for each incidence is scarcely reported. This analysis included 8076 participants of the Nagahama study in Japan (median age 57, 31.0% male) and associations between nocturia and self-reported, sleep-related problems (poor sleep) were evaluated cross-sectionally. Causal effects on each new-onset case were analyzed longitudinally after 5 years. Three models were applied: univariable analysis, adjustment for basic variables (i.e., demographic and lifestyle variables) and full adjustment for basic and clinical variables. The overall prevalences of poor sleep and nocturia were 18.6% and 15.5%, while poor sleep was positively associated with nocturia (OR = 1.85, p < 0.001) and vice versa (OR = 1.90, p < 0.001). Among 6579 good sleep participants, 18.5% developed poor sleep. Baseline nocturia was positively associated with this incident poor sleep (OR = 1.49, p < 0.001, full adjustment). Among 6824 non-nocturia participants, the nocturia incidence was 11.3%. Baseline poor sleep was positively associated with this incident nocturia (OR = 1.26, p = 0.026); such associations were significant only in women (OR = 1.44, p = 0.004) and under-50-year-old groups (OR = 2.82, p < 0.001), after full adjustment. Nocturia and poor sleep associate with each other. Baseline nocturia can induce new-onset poor sleep while baseline poor sleep may induce new-onset nocturia only in women.

## Introduction

Persistent nocturia, waking to urinate during primary sleep periods^1^, is highly prevalent in people aged 60 years or older (over 60%) and is associated with decreased quality of life, fractures, and even increased mortality^2–4^. Chronic insomnia, the inability to fall asleep or maintain sleep, is also common in aged people (~ 10–15% prevalence)^5^ and is associated with morbidity and mortality^6^. Thus, synergy between the two conditions exists as insomniacs may awaken at night due to nocturia and be unable to fall back into sleep. The effect of such cross-sectional relationships in patient interventions for nocturia and insomnia has been reported in numerous epidemiological analyses and several clinical studies^7–14^.

However, as diseases (such as obstructive sleep apnea and chronic heart failure) may also induce both nocturia and insomnia, making causal determinations can be difficult and complicate treatment. For example, benign prostatic hyperplasia or the higher incidence of obstructive sleep apnea in men may contribute to joint nocturia/insomnia morbidity while hormonal imbalances in women may result in variable susceptibility to either or both afflictions^15–17^.

Longitudinal analyses are useful to address issues of association between nocturia and poor sleep. One large-scale study was reported by Araujo et al.^18^ from the BACH (Boston Area Community Health) Survey, consisting of 4144 participants, and showed the bidirectional effects of self-reported, poor sleep-related problems and nocturia. However, currently, no similar, large-scale reports have since explored such longitudinal relationships. Thus, the aim of this study was to complete a longitudinal study on interlocking factors between nocturia and self-reported, sleep-related problems in a large Japanese cohort over 5 years while focusing on gender differences. Here, the relationship between nocturia and self-reported, sleep-related problems at baseline by cross-sectional analysis is demonstrated, also discovering links between new-onset nocturia/baseline self-reported, sleep-related problems and vice versa.

## Participants and methods

The Nagahama study is a longitudinal and population-based health survey set in Nagahama City, Japan and is comprised of a questionnaire survey plus anthropometric, physiological, and biochemical measures of participants aged 30–75 years. The first survey was given to 9764 people from 2008 to 2010 and the follow-up evaluation was performed 5 years later. Participants were excluded based on the following criteria: those who could not attend the follow-up survey, (2) did not complete item 7 in the International Prostate Symptom Score (IPSS) or other questionnaire items needed to evaluate baseline or clinical variables, (3) had undertreatment of hemodialysis or prostatic diseases, (4) were pregnant, (5) or had a treatment history of obstructive sleep apnea (Supplementary Fig. 1). After exclusion, data from 8076 participants were analyzed by cross-sectional analysis (Analysis 1). After excluding individuals with baseline poor sleep, the association between nocturia and new-onset poor sleep was analyzed longitudinally in 6579 participants (Analysis 2). Similarly, after excluding individuals with baseline nocturia, the association of poor sleep and new-onset nocturia was analyzed in 6824 participants (Analysis 3) (Supplementary Fig. 1).

This study was designed in compliance with the Declaration of Helsinki and was approved by the Kyoto University Graduate School and Faculty of Medicine Ethics Committee (No. G278), the Ad Hoc Review Board of the Nagahama Study, and the Nagahama Municipal Review Board of Personal Information Protection. All participants provided written, informed consent to participate and allow their data to be used in the present study.

### Definitions

Nocturia was defined as 2 or more episodes of nocturnal urinary frequency based on IPSS item 7^11,18^. Self-reported, sleep-related problems were assessed based on questionnaires that asked “I can’t sleep well and/or “I take sleeping pills more than once a week. The criteria for defining “poor sleep” was based on the presence of an affirmative answer to either or both questions while “good sleep” was defined as the absence of affirmative answers to both questions. Physical activity was evaluated using the item “Do you spend most of your life sitting or in static activity?”. Alcohol consumption was evaluated as drinking one or more “go”, with one “go” being equivalent to 22 g of ethanol. Smoking was defined by the answer to the question "Do you currently smoke cigarettes?" Overweight or obesity was defined as Body Mass Index ≥ 25 kg/m^2^. The presence of hyperlipidemia was determined based on low-density lipoprotein ≥ 140 mg/dl, high-density lipoprotein < 40 mg/dl, or currently taking antihyperlipidemic medication. Hypertension was determined based on medical history and actual measured values (systemic blood pressure ≥ 140 mmHg or diastolic blood pressure ≥ 90 mmHg). Renal insufficiency was defined as an estimated glomerular filtration rate of less than 60 ml/min/1.73 m^2^ using the formula in the modified Modification of Diet in Renal Disease definition for Japanese people. Hyperglycemia was defined based on medical history, current use of antidiabetic medication, fasting plasma glucose ≥ 126 mg/dl, occasional plasma glucose ≥ 200 mg/dl, and glycated hemoglobin levels (A1c ≥ 6.5%). Obstructive sleep apnea was determined based on medical history. Mental health was assessed by the Mental Health Inventory-5 (MHI-5), a screening questionnaire for anxiety and depressive symptoms. The definition of new-onset nocturia refers to individuals who did not report nocturia at baseline but developed the condition during the 5-year follow-up period. Similarly, new-onset poor sleep refers to individuals who did not report poor sleep at baseline but developed sleep-related problems during the follow-up period.

### Statistical analyses

Factors independently associated with nocturia and poor sleep were assessed using multivariable logistic regression analysis with three models: (1) univariate analysis, (2) adjustment for baseline basic variables (i.e., demographic and lifestyle variables), and (3) adjustment for baseline basic and clinical variables (hyperglycemia, hyperlipidemia, hypertension, renal insufficiency, poor sleep, obstructive sleep apnea, and mental health). A p value less than 0.05 was considered statistically significant. All statistical analyses were performed using the commercially available software package JMP 14.2.0 (SAS, Cary, NC, USA).

## Results

**Figure 1:**
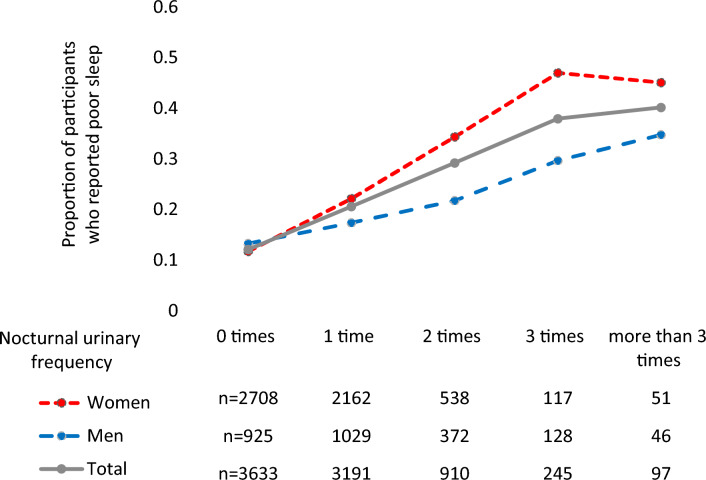
Proportion of participants who reported poor sleep by nocturnal urinary frequency.

The median age of the 8076 participants was 57 years old *in toto*: 2500 male participants (31.0%) had a median age of 60 and 5576 female participants (69.0%) had a median age of 55. The overall prevalence of poor sleep and nocturia were 1497 (18.6%) and 1252 (15.5%), with 437 (17.5%) men and 1060 (19.0%) women suffering from poor sleep and 546 (21.8%) men and 706 (12.7%) women suffering from nocturia (Table 1). The prevalence of poor sleep increased along with the night-time urination frequency in both men and women (Fig. 1).

**Table 1:** Baseline characteristics of participants.

	Men	Women	Total
No. participants (n, %)	2500 (31.0)	5576 (69.0)	8076
Basic variables			
Age, years (median, quartile)	60 (43, 67)	55 (41, 63)	57 (30, 75)
Daily activity (static)	285 (11.4)	395 (7.1)	680 (8.4)
Overweight or obesity (n, %)	667 (26.7)	790 (14.2)	1457 (18.0)
Alcohol intake (n, %)	1227 (45.1)	321 (5.8)	1448 (17.9)
Smoking status (n, %)	738 (29.5)	325 (5.8)	1063 (13.2)
Clinical variables			
BNP (pg/mL, median, quartile)	10.6 (6.0, 18.5)	13.3 (8.2, 21.7)	12.6 (7.6, 20.9)
Hyperlipidemia (n, %)	1290 (51.6)	2372 (43.5)	3662 (45.3)
Hypertension (n, %)	1249 (50.1)	1620 (29.2)	2869 (35.7)
Hyperglycemia (n, %)	177 (7.1)	134 (2.4)	311 (3.9)
Renal insufficiency (n, %)	1639 (65.6)	373 (6.7)	2012 (24.9)
Obstructive sleep apnea (n, %)	290 (11.6)	87 (1.6)	377 (4.7)
Mental health score (median, quartile)	19 (17, 22)	19 (16, 21)	19 (16, 21)
Poor sleep (n, %)	437 (17.5)	1060 (19.0)	1497 (18.6)
Nocturia (n, %)	546 (21.8)	706 (12.7)	1252 (15.5)

*Analysis 1: A Cross-sectional Analysis of Association between Poor Sleep and Nocturia*. In the cross-sectional multivariable analysis, poor sleep was positively associated with nocturia (odds ratio (OR) 1.85, p < 0.001) and nocturia was also positively associated with poor sleep (OR 1.90, p < 0.001) in total, both adjusted for basic and clinical variables. After gender stratification, these positive associations were observed in men (OR 1.43, p < 0.001 and OR 1.47, p = 0.003) and in women (OR 2.19, p < 0.001 and OR 2.21, p < 0.001) (Supplementary Table 1).

**Figure 2:**
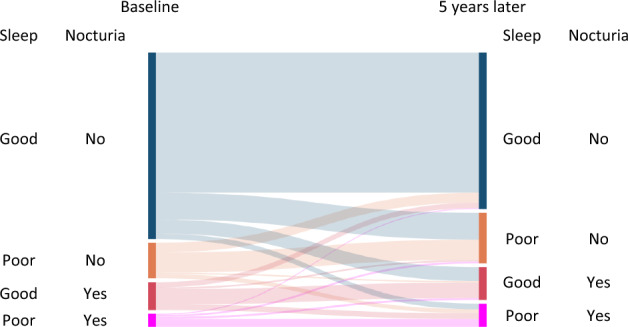
Sanky diagram visualizing the prevalence flow of nocturia and poor sleep among participants.

*Analysis 2: Nocturia and New-onset Poor Sleep*. The prevalence flow of nocturia and poor sleep from baseline to 5 years later is shown in Fig. 2. Among 6579 good sleep participants, new-onset poor sleep occurrences at 5-year follow-up were 1220 (18.5%). Nocturia at baseline was significantly and strongly associated with new-onset poor sleep with an OR of 1.70 (p < 0.001) unadjusted, 1.55 (p < 0.001) adjusted for basic variables, and 1.49 (p < 0.001) adjusted for basic and clinical variables (Table 2). Stratified by gender, new-onset poor sleep occurred in 17.8% of men and in 18.9% of women while nocturia at baseline was significantly associated with new-onset poor sleep with an OR of 1.73 (p < 0.001) in men and 1.41 (p = 0.004) in women, adjusted for basic and clinical variables (Table 2). Stratified by age (over/under 50 years old), nocturia at baseline was significantly associated with new-onset poor sleep in both groups with an OR of 1.46 (p < 0.001) and 1.84 (p = 0.019), respectively, adjusted for basic and clinical variables (Supplementary Table 2). Gender differences were clearly observed in the under-50 cohort with an interaction of gender and incidence of poor sleep (p = 0.001). The OR was 4.56 in men (p < 0.001) and 1.09 in women (p = 0.83, not significant), adjusted for basic and clinical variables) while the baseline prevalence of nocturia in women was very low (3.0%, 55/1861, Supplementary Table 2).

**Table 2:** Incidence of poor sleep with and without baseline nocturia by logistic regression analysis in this longitudinal study.

Men and women			
	Nocturia (n = 854)	No nocturia (n = 5725)	*p* value
Incident poor sleep (n, %)	225 (26.4)	995 (17.4)	
Crude model (95% CI)	1.70 (1.44–2.01)	1.00 (Reference)	< 0.001
Adjusted model (95% CI)			
Basic variables	1.55 (1.30–1.85)	1.00 (Reference)	< 0.001
Basic and clinical variables	1.49 (1.25–1.79)	1.00 (Reference)	< 0.001

Men			
	Nocturia (n = 411)	No nocturia (n = 1652)	*p* value
Incident poor sleep (n, %)	100 (24.3)	267 (16.2)	
Crude model (95% CI)	1.67 (1.29–2.17)	1.00 (Reference)	< 0.001
Adjusted model (95% CI)			
Basic variables	1.76 (1.33–2.34)	1.00 (Reference)	< 0.001
Basic and clinical variables	1.73 (1.30–2.30)	1.00 (Reference)	< 0.001

Women			
	Nocturia (n = 443)	No nocturia (n = 4073)	*p* value
Incident poor sleep (n, %)	125 (28.2)	728 (17.9)	
Crude model (95% CI)	1.81 (1.45–2.25)	1.00 (Reference)	< 0.001
Adjusted model (95% CI)			
Basic variables	1.48 (1.18–1.87)	1.00 (Reference)	< 0.001
Basic and clinical variables	1.41 (1.12–1.79)	1.00 (Reference)	0.004

*Analysis 3: Poor sleep and New-onset Nocturia*. Among 6824 non-nocturia participants at baseline, the prevalence of new-onset nocturia at follow-up was 772 (11.3%). Poor sleep at baseline was significantly associated with the new-onset of nocturia with an OR of 1.60 (p < 0.001) unadjusted, 1.34 (p = 0.003) adjusted for basic variables, and 1.26 (p = 0.026) adjusted for basic and clinical variables (Table 3). Gender differences were analyzed and new-onset nocturia occurred in 17.2% of men and in 9.0% of women with an interaction between gender and incident nocturia (p = 0.046). Poor sleep at baseline was not significantly associated with new-onset nocturia (OR of 1.02, p = 0.92) in men but significantly associated in women (OR = 1.44, p = 0.004) after adjustment for basic and clinical variables (Table 3). Of note, age stratification (over/under 50 years) found an interaction with incident nocturia (p < 0.001). Poor sleep at baseline was significantly associated with new-onset of nocturia in the under-50 group, with an odds ratio of 2.82 (p < 0.001) in total, 2.96 (p = 0.029) in men, and 2.74 (p < 0.001) in women, after adjustment for basic and clinical variables. This effect was not seen in the over-50 group (Supplementary Table 3).

**Table 3:** Incidence of nocturia with and without baseline poor sleep by logistic regression analysis in this longitudinal study.

	Poor sleep (n = 1099)	Good sleep (n = 5725)	*p* value
Incident nocturia (n, %)	173 (15.7)	599 (10.5)	
Crude model (95% CI)	1.60 (1.33–1.92)	1.00 (Reference)	< 0.001
Adjusted model (95% CI)			
Basic variables	1.34 (1.11–1.63)	1.00 (Reference)	0.003
Basic and clinical variables	1.26 (1.03–1.54)	1.00 (Reference)	0.026

Men			
	Poor sleep (n = 302)	Good sleep (n = 1652)	*p* value
Incident nocturia (n, %)	61 (20.2)	274 (16.6)	
Crude model (95% CI)	1.27 (0.93–1.73)	1.00 (Reference)	0.13
Adjusted model (95% CI)			
Basic variables	1.10 (0.79–1.53)	1.00 (Reference)	0.59
Basic and clinical variables	1.02 (0.72–1.44)	1.00 (Reference)	0.92

Women			
	Poor sleep (n = 797)	Good sleep (n = 4073)	*p* value
Incident nocturia (n, %)	112 (14.1)	325 (8.0)	
Crude model (95% CI)	1.89 (1.50–2.38)	1.00 (Reference)	< 0.001
Adjusted model (95% CI)			
Basic variables	1.51 (1.19–1.92)	1.00 (Reference)	< 0.001
Basic and clinical variables	1.44 (1.12–1.84)	1.00 (Reference)	0.004

## Discussion

In the present cohort study of a relatively healthy population, nocturia and poor sleep were significantly associated with each other in a cross-sectional analysis. In a longitudinal analysis, nocturia was clearly a significant risk for incident poor sleep while poor sleep was a statistically significant but weak risk for incident nocturia.

The results of the cross-sectional analysis showed that associations between nocturia and poor sleep were highly dependent on gender, an effect seen in other studies^19^. Men, who are prone to urological issues (such as prostate enlargement) as they age, may find these causes more contributive than poor sleep and tend to fall asleep quickly even if they have nocturia^12^. On the other hand, due to hormonal issues, women may be more susceptible to poor sleep in mid-life, leading to nocturia due to wakefulness^20^. Thus, any studies evaluating relationships between poor sleep and nocturia should be inclusive of both sexes and stratify results by sex whenever possible.

The results of the longitudinal analysis showed that both men and women with nocturia were at risk for new-onset of poor sleep at 5-year follow-up in accordance with previous reports^18,21^. Obviously, waking at night to urinate will interrupt sleep cycles and disrupt the circadian rhythm due to light exposure, possibly inducing poor sleep over the long term^22–24^. Regarding the risk of nocturia causing poor sleep in the under-50 cohort, about half of men with nocturia developed poor sleep but women did not experience this to the same degree. Thus, these data suggest men under 50 with nocturia as a special risk population for poor sleep pathogenesis.

The longitudinal analysis also showed a somewhat positive association between poor sleep and incident nocturia in all participants, with a p-value slightly below 0.05 and a relatively low odds ratio. Of note, there was no such association in men and the risk was only in women after gender stratification. In women, the relatively strong association between poor sleep and nocturia in the cross-sectional analysis of this study may explain the significant influence on incident nocturia over the 5-year study period. Other factors may also be as responsible for this since causes of nocturia in men over 50 years of age are diverse, including benign prostatic hyperplasia and nocturnal polyuria^25^. As such, using 50 years of age as a stratification cutoff was useful in eliminating some of these causes for younger-middle-aged men in the analysis. As a consequence, the risk was evident in those under 50. Since only a few factors in younger people contribute to the incidence of nocturia (other than poor sleep), the true effect of poor sleep on incident nocturia may be more evident in these younger populations.

Taken together, the findings of this study suggest that interventions to improve nocturia may be preventative against poor sleep. Appropriate guidance and medications should be provided while behavior and lifestyle modifications, such as fluid/caffeine restriction and leg elevation in the evening, stockings for peripheral edema, pelvic floor muscle training, delayed voiding, and urge suppression^26^ may be useful. Additionally, clear circadian boundaries in activities, such as exercise in the evening^27^, taking a bath 1–2 h before bedtime^28^, and light exposure in the morning^29^ could reduce nocturnal urination and may prevent incident poor sleep and disruption of deep sleep, thereby extending healthy life expectancy. Desmopressin administration to patients with nocturnal polyuria has additionally been shown to significantly improve both nocturia and the duration of the first sleep period^30^. Proactive interventions for poor sleep caused by stress, depression, alcohol consumption, and lifestyle disturbances would also be desirable in terms of prevention of nocturia.

This study has several limitations. Firstly, for poor sleep, only sleep quality (yes/no) and presence/absence of sleeping pills were analyzed while the degree and classification of sleep disorders were not evaluated. Therefore, the causal association between the degree of poor sleep and nocturnal urinary frequency remains unknown. Follow-up is ongoing with more quantifiable evaluation methods and future studies are expected. Secondly, although participants with a history of dialysis, prostate disease, or undergoing treatment for obstructive sleep apnea (which may be directly related to nocturia) were excluded, therapeutic interventions for nocturia and poor sleep during the 5-year course of the study were not evaluated. Thirdly, selection biases may exist in this study. The cross-sectional analysis excluded participants who were not available for follow-up to be evaluated in the same population as the longitudinal analysis. Subgroup analyses were performed, stratified by gender age cutoffs from a clinical view, while interactions were evaluated in the multivariable analyses. The prevalence of nocturia itself is low among participants younger than 50 years of age. Finally, this is an epidemiological study and the mechanism of the associations between nocturia and poor sleep is not known; however, the gender and age differences highlighted in this study may shed light on approaches to fully understanding such mechanisms.

## Conclusions

Nocturia and self-reported, sleep-related problems were closely related to each other, with differences in their effects depending on gender and age. Nocturia was clearly associated with risk of developing self-reported, sleep-related problems in both men and women. On the other hand, self-reported, sleep-related problems were associated with the risk of nocturia onset in women only.

## Supplementary Information

Supplementary Information.

## Data Availability

The datasets generated during and/or analysed during the current study are not publicly available due to the cohort data of the Nagahama study group but are available from the corresponding author on reasonable request.
