# Supplementary material for: Risk analyses of nocturia on incident poor sleep and vice versa: the Nagahama study
Source: Sci Rep. 2023 Jun 11;13:9495. doi: 10.1038/s41598-023-36707-y (PMC10258194; doi:10.1038/s41598-023-36707-y)
Supplement: Supplementary file 1 — Supplementary Information. [file 41598_2023_36707_MOESM1_ESM.pdf]

Supplementary Information for

Risk analyses of nocturia on incident poor sleep and vice versa: The Nagahama Study

Hiromitsu Negoro[1,2], Kazuya Setoh[3,4], Arinobu Fukunaga[2], Takahisa Kawaguchi[3], Satoshi Funada[2], Takayuki Yoshino[1,2], Koji Yoshimura[5], Bryan J. Mathis[6], Yasuharu Tabara[3,4], Fumihiko Matsuda[3], Osamu Ogawa[2], Takashi Kobayashi[2]. on behalf of the Nagahama Study Group

1 Department of Urology, University of Tsukuba, Ibaraki, Japan
2 Department of Urology, Kyoto University Graduate School of Medicine, Kyoto, Japan
3 Center for Genomic Medicine, Kyoto University Graduate School of Medicine, Kyoto, Japan
4 Graduate School of Public Health, Shizuoka Graduate University of Public Health, Aoi-ku, Shizuoka, Japan
5 Department of Urology, Shizuoka General Hospital, Shizuoka, Japan
6 International Medical Center, University of Tsukuba Affiliated Hospital, Ibaraki, Japan.

Correspondence should be addressed to Hiromitsu Negoro (E-mail: hnegoro@md.tsukuba.ac.jp)

Supplementary Information includes Supplementary Figure 1 and Table 1 to 3

Supplementary Figure 1 A flow diagram of the participants

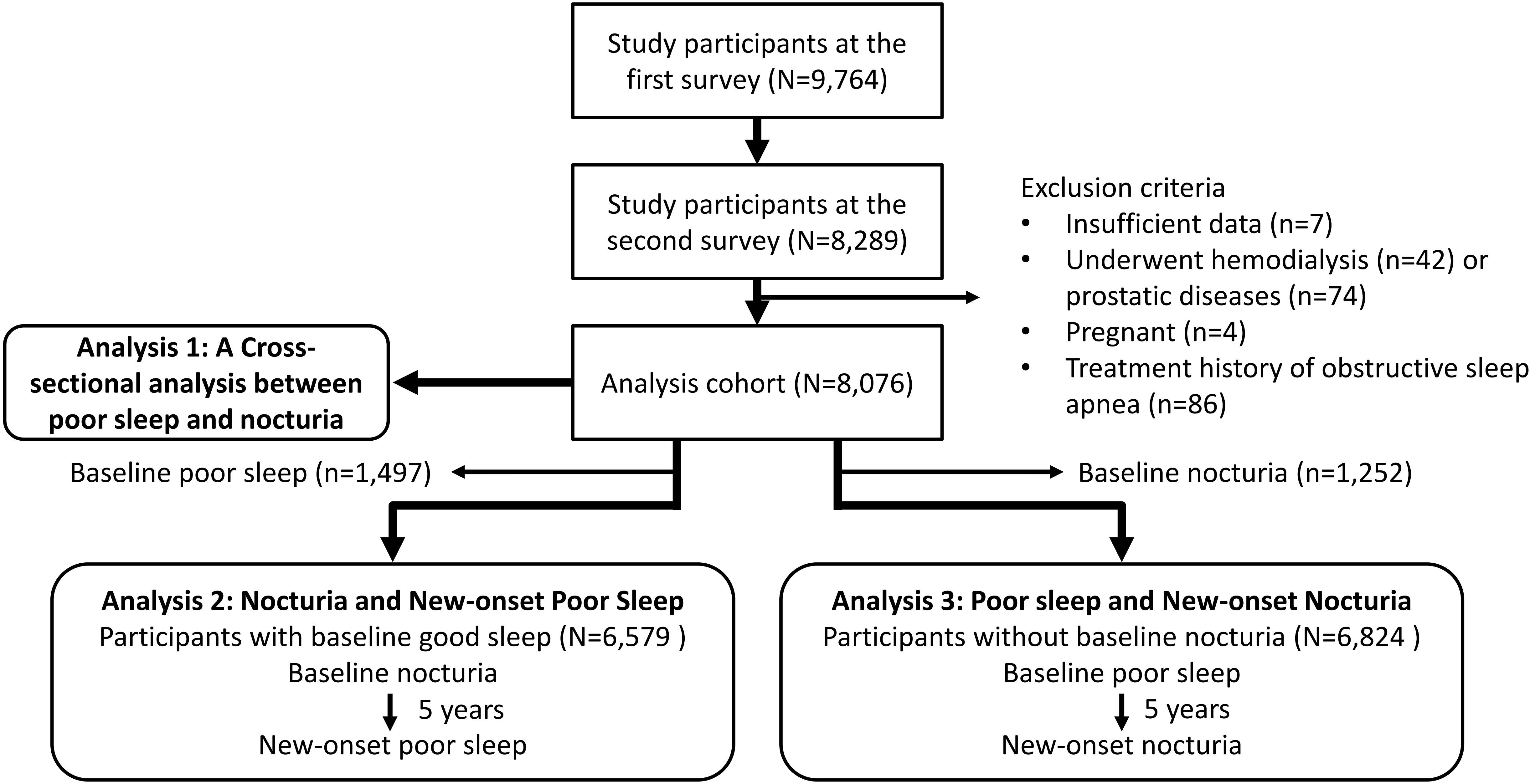

Supplementary Table 1 Association between poor sleep and nocturia by logistic regression analysis in the cross-sectional study

Men and women

	Poor sleep (n=1,497)	Good sleep (n=6,579)	*P* value
Nocturia (n, %)	398 (26.6)	854 (13.0)	
Crude model (95% CI)	2.43 (2.12-2.78)	1.00 (Reference)	<0.001
Adjusted model (95% CI)			
Basic variables	2.03 (1.76-2.35)	1.00 (Reference)	<0.001
Basic and clinical variables	1.85 (1.59-2.14)	1.00 (Reference)	<0.001

	Nocturia (n=1,252)	No nocturia (n=6,824)	*P* value
Poor sleep (n, %)	398 (31.8)	1,099 (16.1)	
Crude model (95% CI)	2.42 (2.12-2.78)	1.00 (Reference)	<0.001
Adjusted model (95% CI)			
Basic variables	2.09 (1.81-2.42)	1.00 (Reference)	<0.001
Basic and clinical variables	1.90 (1.64-2.21)	1.00 (Reference)	<0.001

Men

	Poor sleep (n=437)	Good sleep (n=2,063)	*P* value
Nocturia (n, %)	135 (30.1)	411 (19.9)	
Crude model (95% CI)	1.77 (1.41-2.23)	1.00 (Reference)	<0.001
Adjusted model (95% CI)			
Basic variables	1.61 (1.25-2.06)	1.00 (Reference)	<0.001
Basic and clinical variables	1.43 (1.10-1.84)	1.00 (Reference)	<0.001

	Nocturia (n=546)	No nocturia (n=1,954)	*P* value
Poor sleep (n, %)	135 (24.7)	302 (15.5)	
Crude model (95% CI)	1.80 (1.42-2.26)	1.00 (Reference)	<0.001
Adjusted model (95% CI)			
Basic variables	1.63 (1.27-2.09)	1.00 (Reference)	<0.001
Basic and clinical variables	1.47 (1.14-1.90)	1.00 (Reference)	0.003

Women

	Poor sleep (n=1,060)	Good sleep (n=4,516)	*P* value
Nocturia (n, %)	263 (24.8)	443 (9.8)	
Crude model (95% CI)	3.06 (2.58-3.62)	1.00 (Reference)	<0.001
Adjusted model (95% CI)			
Basic variables	2.42 (2.02-2.89)	1.00 (Reference)	<0.001
Basic and clinical variables	2.19 (1.82-2.64)	1.00 (Reference)	<0.001

	Nocturia (n=706)	No nocturia (n=4,870)	*P* value
Poor sleep (n, %)	263 (37.3)	797 (16.4)	
Crude model (95% CI)	3.03 (2.56-3.60)	1.00 (Reference)	<0.001
Adjusted model (95% CI)			
Basic variables	2.44 (2.04-2.92)	1.00 (Reference)	<0.001
Basic and clinical variables	2.21 (1.83-2.66)	1.00 (Reference)	<0.001

Supplementary Table 2 Incidence of poor sleep with and without baseline nocturia by logistic regression analysis in the longitudinal study, stratified by age at 50 years.

Participants aged 50 years old and over

Men and women

	Nocturia (n=770)	No nocturia (n=3,273)	*P* value
Incident poor sleep (n, %)	202 (26.2)	639 (19.5)	
Crude model (95% CI)	1.47 (1.22-1.76)	1.00 (Reference)	<0.001
Adjusted model (95% CI)			
Basic variables	1.52 (1.26-1.85)	1.00 (Reference)	<0.001
Basic and clinical variables	1.46 (1.20-1.78)	1.00 (Reference)	<0.001

Men

	Nocturia (n=382)	No nocturia (n=1,006)	*P* value
Incident poor sleep (n, %)	86 (22.5)	163 (16.2)	
Crude model (95% CI)	1.50 (1.12-2.01)	1.00 (Reference)	0.006
Adjusted model (95% CI)			
Basic variables	1.49 (1.09-2.02)	1.00 (Reference)	0.011
Basic and clinical variables	1.47 (1.07-2.00)	1.00 (Reference)	0.017

Women

	Nocturia (n=388)	No nocturia (n=2,267)	*P* value
Incident poor sleep (n, %)	116 (29.9)	476 (21.0)	
Crude model (95% CI)	1.60 (1.26-2.04)	1.00 (Reference)	<0.001
Adjusted model (95% CI)			
Basic variables	1.55 (1.21-1.99)	1.00 (Reference)	<0.001
Basic and clinical variables	1.47 (1.14-1.89)	1.00 (Reference)	0.003

Participants aged less than 50 years old

Men and women

	Nocturia (n=84)	No nocturia (n=2,452)	*P* value
Incident poor sleep (n, %)	23 (27.4)	356 (14.5)	
Crude model (95% CI)	2.22 (1.36-3.63)	1.00 (Reference)	0.002
Adjusted model (95% CI)			
Basic variables	2.13 (1.30-3.50)	1.00 (Reference)	0.003
Basic and clinical variables	1.84 (1.11-3.06)	1.00 (Reference)	0.019

Men

	Nocturia (n=29)	No nocturia (n=646)	*P* value
Incident poor sleep (n, %)	14 (48.3)	104 (16.1)	
Crude model (95% CI)	4.86 (2.27-10.38)	1.00 (Reference)	<0.001
Adjusted model (95% CI)			
Basic variables	5.01 (2.32-10.81)	1.00 (Reference)	<0.001
Basic and clinical variables	4.56 (2.05-10.16)	1.00 (Reference)	<0.001

Women

	Nocturia (n=55)	No nocturia (n=1,806)	*P* value
Incident poor sleep (n, %)	9 (16.4)	252 (14.0)	
Crude model (95% CI)	1.21 (0.58-2.50)	1.00 (Reference)	0.61
Adjusted model (95% CI)			
Basic variables	1.21 (0.58-2.51)	1.00 (Reference)	0.62
Basic and clinical variables	1.09 (0.51-2.30)	1.00 (Reference)	0.83

Supplementary Table 3 Incidence of nocturia with and without baseline poor sleep by logistic regression analysis in the longitudinal study, stratified by age at 50 years.

Participants aged 50 years old and over

Men and women

	Poor sleep (n=753)	Good sleep (n=3,273)	*P* value
Incident nocturia (n, %)	148 (19.7)	540 (16.5)	
Crude model (95% CI)	1.24 (1.01-1.51)	1.00 (Reference)	0.038
Adjusted model (95% CI)			
Basic variables	1.15 (0.94-1.41)	1.00 (Reference)	0.19
Basic and clinical variables	1.10 (0.88-1.36)	1.00 (Reference)	0.39

Men

	Poor sleep (n=202)	Good sleep (n=1,006)	*P* value
Incident nocturia (n, %)	53 (26.2)	257 (25.6)	
Crude model (95% CI)	1.04 (0.73-1.46)	1.00 (Reference)	0.84
Adjusted model (95% CI)			
Basic variables	0.96 (0.67-1.37)	1.00 (Reference)	0.81
Basic and clinical variables	0.90 (0.62-1.30)	1.00 (Reference)	0.57

Women

	Poor sleep (n=551)	Good sleep (n=2,267)	*P* value
Incident nocturia (n, %)	95 (17.2)	283 (12.5)	
Crude model (95% CI)	1.46 (1.13-1.88)	1.00 (Reference)	0.003
Adjusted model (95% CI)			
Basic variables	1.34 (1.03-1.73)	1.00 (Reference)	0.028
Basic and clinical variables	1.26 (0.97-1.66)	1.00 (Reference)	0.088

Participants aged less than 50 years old

Men and women

	Poor sleep (n=346)	Good sleep (n=2,452)	*P* value
Incident nocturia (n, %)	25 (7.2)	59 (2.4)	
Crude model (95% CI)	3.16 (1.95-5.12)	1.00 (Reference)	<0.001
Adjusted model (95% CI)			
Basic variables	3.13 (1.93-5.08)	1.00 (Reference)	<0.001
Basic and clinical variables	2.82 (1.68-4.74)	1.00 (Reference)	<0.001

Men

	Poor sleep (n=100)	Good sleep (n=646)	*P* value
Incident nocturia (n, %)	8 (8.0)	17 (2.6)	
Crude model (95% CI)	3.21 (1.35-7.67)	1.00 (Reference)	0.008
Adjusted model (95% CI)			
Basic variables	3.34 (1.39-8.04)	1.00 (Reference)	0.007
Basic and clinical variables	2.96 (1.12-7.85)	1.00 (Reference)	0.029

Women

	Poor sleep (n=246)	Good sleep (n=1,806)	*P* value
Incident nocturia (n, %)	17 (6.9)	42 (2.3)	
Crude model (95% CI)	3.12 (1.75-5.57)	1.00 (Reference)	<0.001
Adjusted model (95% CI)			
Basic variables	3.02 (1.69-5.42)	1.00 (Reference)	<0.001
Basic and clinical variables	2.74 (1.48-5.10)	1.00 (Reference)	<0.001
